# Supplementary material for: Chromothripsis during telomere crisis is independent of NHEJ, and consistent with a replicative origin
Source: Genome Res. 2019 May;29(5):737–49. doi: 10.1101/gr.240705.118 (PMC6499312; doi:10.1101/gr.240705.118)
Supplement: Supplemental Material [file supp_gr.240705.118_Supplemental_file_1.zip › contigs/annotated_contigs/DB67/contig.2.DB67_length_332_mean_cov_9.94578313253.docx]

**DB67_length_332_mean_cov_9.94578313253**

```
ATTCATATTACCTCTTGATAAGTCCACAAGAGAATGGCTCCCTAGTTTTTATCGGAACTTGGAATTATTTTATTTCCTCTTGGTAATGA
 >chr15:43660753-43660978 - E=3e-124 p=0e+00
TGATAGTATAGGTCATTATTTTCTAGATAAAAATTCTTTTCTATACTTGAAGGCACATCTTCCTTTTGGTTGTTTTCCATCTTCAAATT

AAACTATATTTGTTACCTTAAGCTTTCCCCCAG|AAGATCCATTCTTT|AAACCTAAGTTTCTGTTGCTCTCTATTGGAACCTTTAAGT
                                  >chr15:43660320-43660441 - E=2e-61
TCTCCAAATATCATGTTAATTGTGGGGAACTAAATTATGTTTATTATTTTCCTGAAAGTCTGTTAGA
```
